# Deeply Infiltrating Endometriosis (DIE) No More: Dienogest as a Game Changer in Rectovaginal Endometriosis

**DOI:** 10.7759/cureus.85165

**Published:** 2025-06-01

**Authors:** Amanda Wei Mun Tan, Xin Yi Ho, Jill Cheng Sim Lee

**Affiliations:** 1 Obstetrics and Gynaecology, KK Women's and Children's Hospital, Singapore, SGP; 2 Urogynaecology, KK Women’s and Children’s Hospital, Singapore, SGP

**Keywords:** deeply infiltrating endometriosis, dienogest, endometriosis, rectovaginal, vaginal mass

## Abstract

Rectovaginal endometriosis remains a therapeutic enigma due to its relatively low incidence and widely differing opinions on the standard of its treatment. Dienogest is a relatively novel therapy for endometriosis and presents major therapeutic potential in the treatment of deeply infiltrating endometriosis. This is the case of a 36-year-old woman who presented to the Urogynaecology clinic with postcoital bleeding and a vaginal mass. She had a history of open myomectomy, removal of rectovaginal cysts, and endometriotic deposits from the Pouch of Douglas one year earlier, with no treatment from then until this presentation. Speculum examination revealed a 3 cm tongue-like firm mass arising from the posterior fornix with contact bleeding, and pelvic ultrasonography confirmed a 3.5 x 2.4 x 1.9 cm mass posterior to the cervix. The biopsy-proven deeply infiltrating endometriosis mass in the posterior fornix and pouch of Douglas responded well to oral Dienogest treatment. After three months of treatment, the patient was symptom-free, and the mass on rectovaginal examination had shrunk to 1 cm. The endometriosis deposits were clearly demonstrated to be in remission during rectovaginal examination and pelvic ultrasonography after nine months of therapy. The patient experienced minimal side effects on Dienogest, namely prolonged menstrual spotting, which also resolved after nine months of therapy. This is the first reported case of Dienogest successfully shrinking rectovaginal deeply infiltrating endometriosis from 3 cm to undetectable on ultrasound and vaginal examination. This case demonstrates the success of Dienogest as a therapeutic modality for rectovaginal endometriosis, highlighting its promising potential as a standard treatment approach in the long-term management of the relapsing, chronic, and debilitating nature of endometriosis.

## Introduction

Vaginal neoplasms that belong to the group of rectovaginal deeply infiltrating endometriosis (DIE) may appear intimidating but are not malignant and may not even necessitate surgical resection with appropriate treatment.

Endometriosis, typically a disease in women of reproductive age, is defined as the presence of endometrial-like tissue outside the uterus that responds to hormonal stimulation. It can be clinically divided into three distinct forms: superficial (peritoneal/serosal), ovarian, and deep endometriosis [1]. Manifestation of endometriosis located more than 5 mm below the peritoneum is called DIE [1], and it can affect the rectovaginal septum, rectosigmoid colon, uterosacral ligament, bladder, and ureters [2]. These lesions, under the influence of oestrogen, can cause chronic inflammation, consequently forming scar tissue and adhesions [2]. The presentation of endometriosis is variable. Classic symptoms include chronic pelvic pain, dysmenorrhoea, dyspareunia, and infertility. Cyclical changes in bowel and bladder among other symptoms can also arise depending on the sites infiltrated by the disease.

The condition is characterized by a progressive disease course with worsening symptoms if not appropriately managed. Women with endometriosis usually experience endometriosis-related pain, infertility, or both [1]. Minimizing disease progression, alleviating symptoms such as pain, reducing endometriotic lesions, and improving the quality of life are therefore the cornerstones of endometriosis management. Currently, treatment for endometriosis is categorized into medical (analgesia, hormonal medications) and surgical interventions. Preferred treatment regimens, however, remain contentious, especially for DIE, due to its multifocal presentation and nature of deep infiltrating nodules that can be surgically challenging [3]. This necessitates the establishment of a long-term treatment algorithm to individualize management and achieve the aforementioned goals of treatment, avoiding recurrent surgical interventions [4].

Dienogest, a fourth-generation progestin, is a selective progesterone receptor agonist that inhibits systemic gonadotrophin secretion and has no androgenic or estrogenic activity. Its antiproliferative and antiangiogenic properties differentiate it from other progestins in the same class. Dienogest is a relatively novel treatment approach that has in recent years established itself as part of the standard medical management of endometriosis. Its effectiveness, safety, and tolerability profile as a long-term management strategy for endometriosis in routine clinical practice are increasingly being researched on a large scale, as shown in the VIPOS study [5]. Despite its proven efficacy in clinical trials, data regarding its long-term therapeutic use, especially in Asian populations, are limited. This report describes a success story of rectovaginal endometriosis treated with Dienogest and its well-tolerated side effects.

## Case presentation

A 36-year-old woman (gravida 2, para 2) presented to the Urogynaecology Clinic with postcoital bleeding and a vaginal mass in June 2020. Previously, an MRI of the abdomen and pelvis done in March 2017 demonstrated a large uterine fibroid, bilateral endometriotic ovarian cysts, with no deep infiltrating lesions. She underwent open myomectomy, removal of rectovaginal cysts, and endometriotic deposits in April 2017. Subsequently, she was treated with the gonadotropin-releasing hormone agonist Leuprolide Depot (Lucrin® 11.25 mg) from March to October 2017 and levonorgestrel-releasing intrauterine system (Mirena) for five months from April to September 2018 (removed due to malposition). She has no other known medical or surgical history. Her menstruation was regular with normal flow, and her cervical smears were up to date and normal.

**Figure 1:**
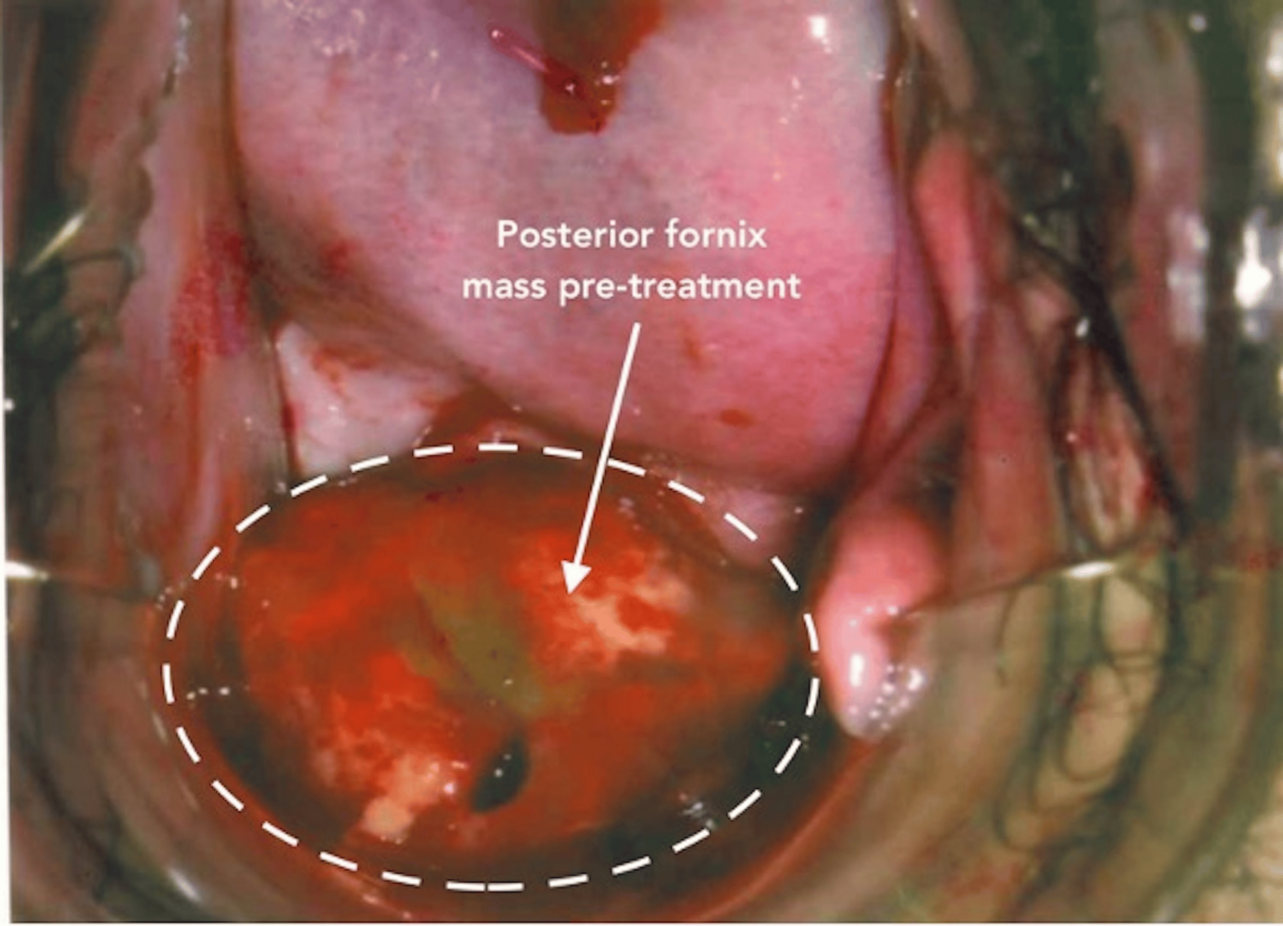
A 3.5 x 2.4 x 1.9 cm mass arising from the posterior fornix during rectovaginal examination, showing contact bleeding before Dienogest treatment.

On representation in June 2020, she had mild dysmenorrhoea and postcoital bleeding for six months, with otherwise regular menstruation. Rectovaginal examination revealed a 3 cm tongue-like firm mass arising from the posterior fornix with contact bleeding (Figure 1). No other endometriotic deposits were noted on ultrasound.

**Figure 2:**
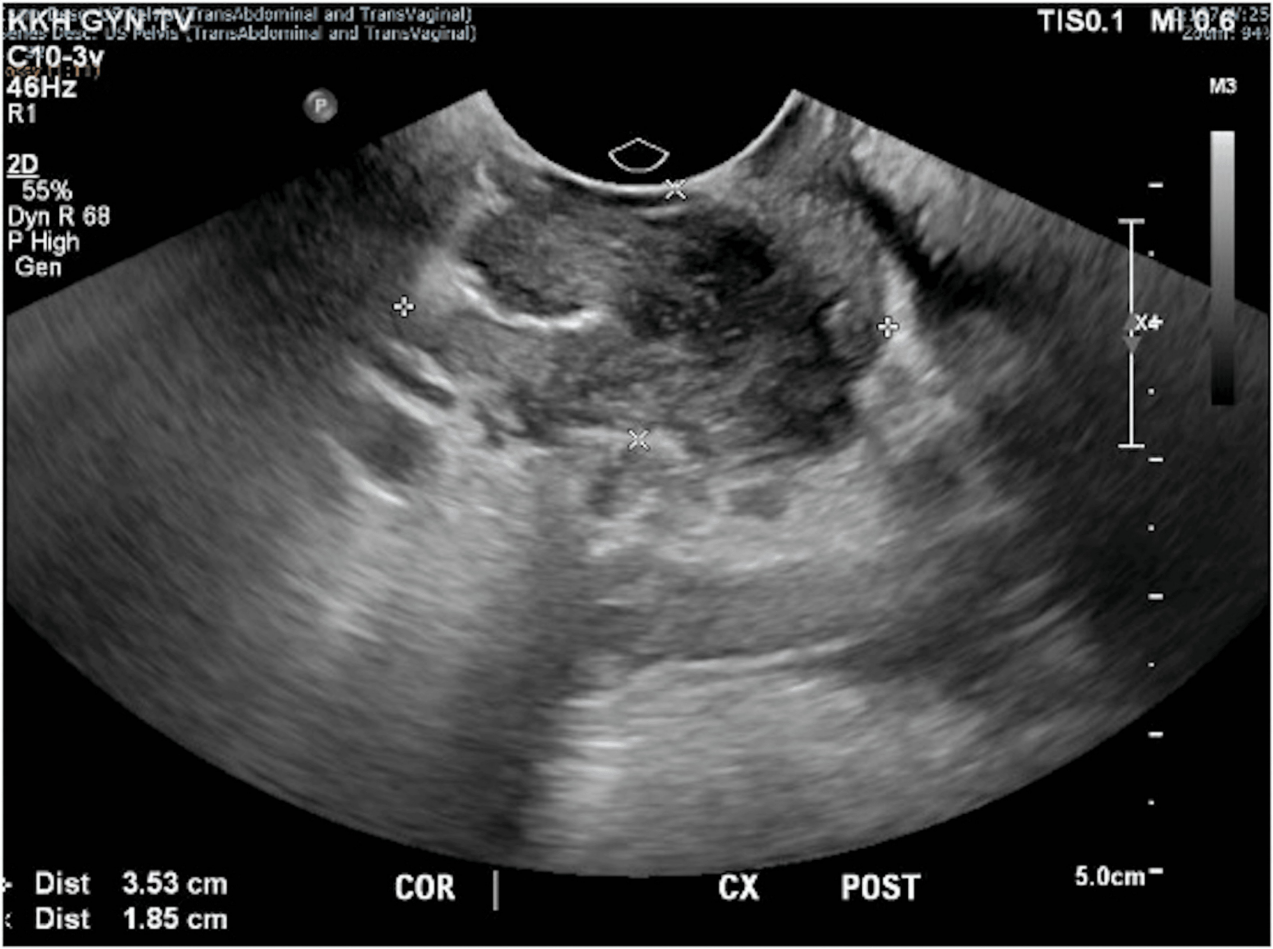
Coronal view of the posterior cervix on transvaginal ultrasonography showing a heterogeneous structure with increased vascularity measuring 3.5 x 2.4 x 1.9 cm posterior to the cervix.

**Figure 3:**
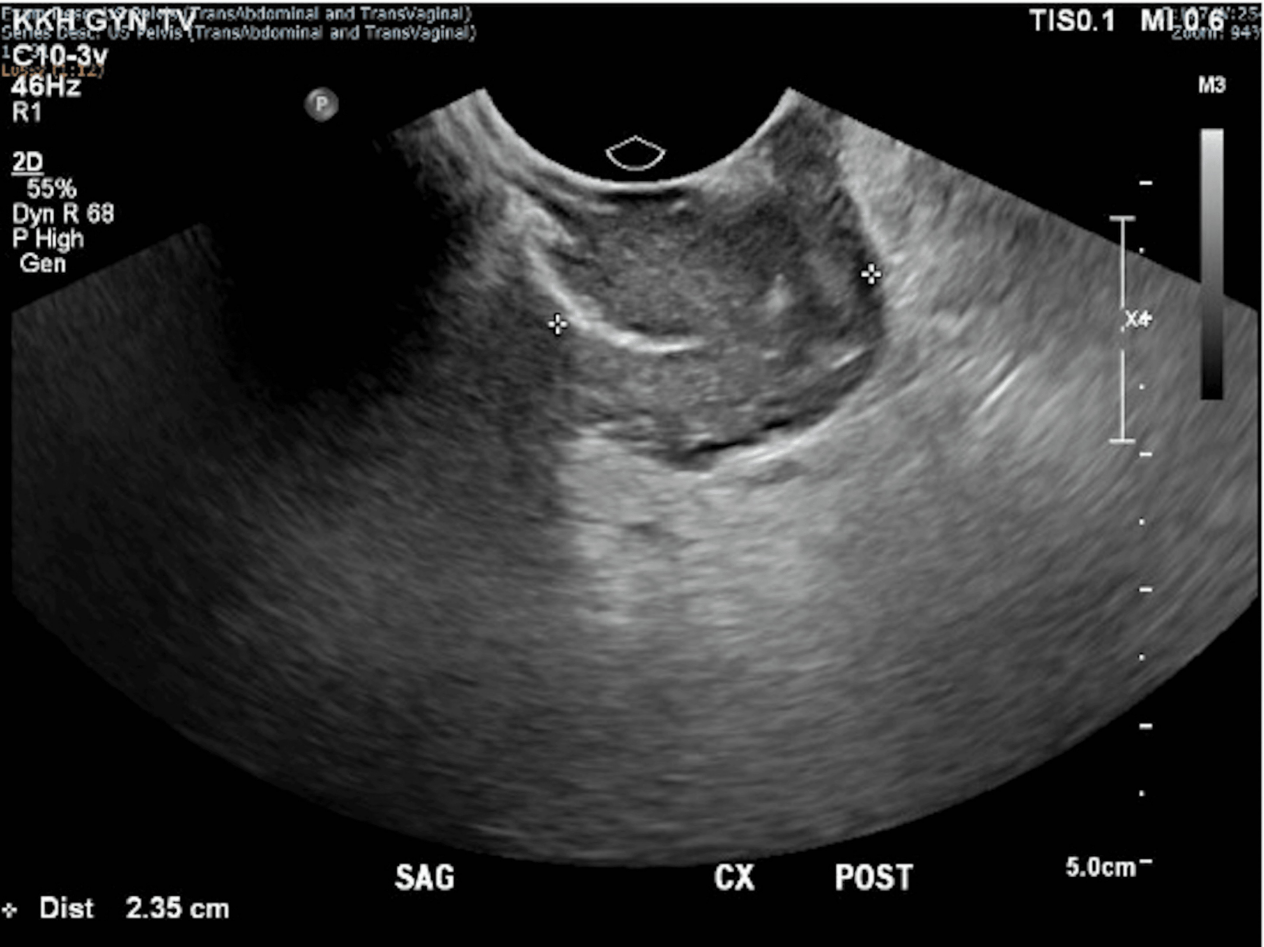
Sagittal view of the posterior cervix on transvaginal ultrasonography showing a heterogeneous structure with increased vascularity measuring 3.5 x 2.4 x 1.9 cm posterior to the cervix.

The mass was clearly seen on pelvic ultrasonography, measuring 3.5 x 2.4 x 1.9 cm posterior to the cervix (Figures 2, 3). Biopsy of the mass histologically confirmed DIE with no overt malignancy in the posterior fornix and Pouch of Douglas. A thorough discussion on management options was conducted with the patient, including conservative surveillance of DIE, oral hormonal medications such as gonadotrophin-releasing hormone agonist and Dienogest, and a repeat surgery (total hysterectomy, bilateral salpingectomy keep in view oophorectomy, with excision of DIE), as the patient had completed her family. The patient was keen to attempt oral Dienogest 2 mg daily, which was started in June 2020.

**Figure 4:**
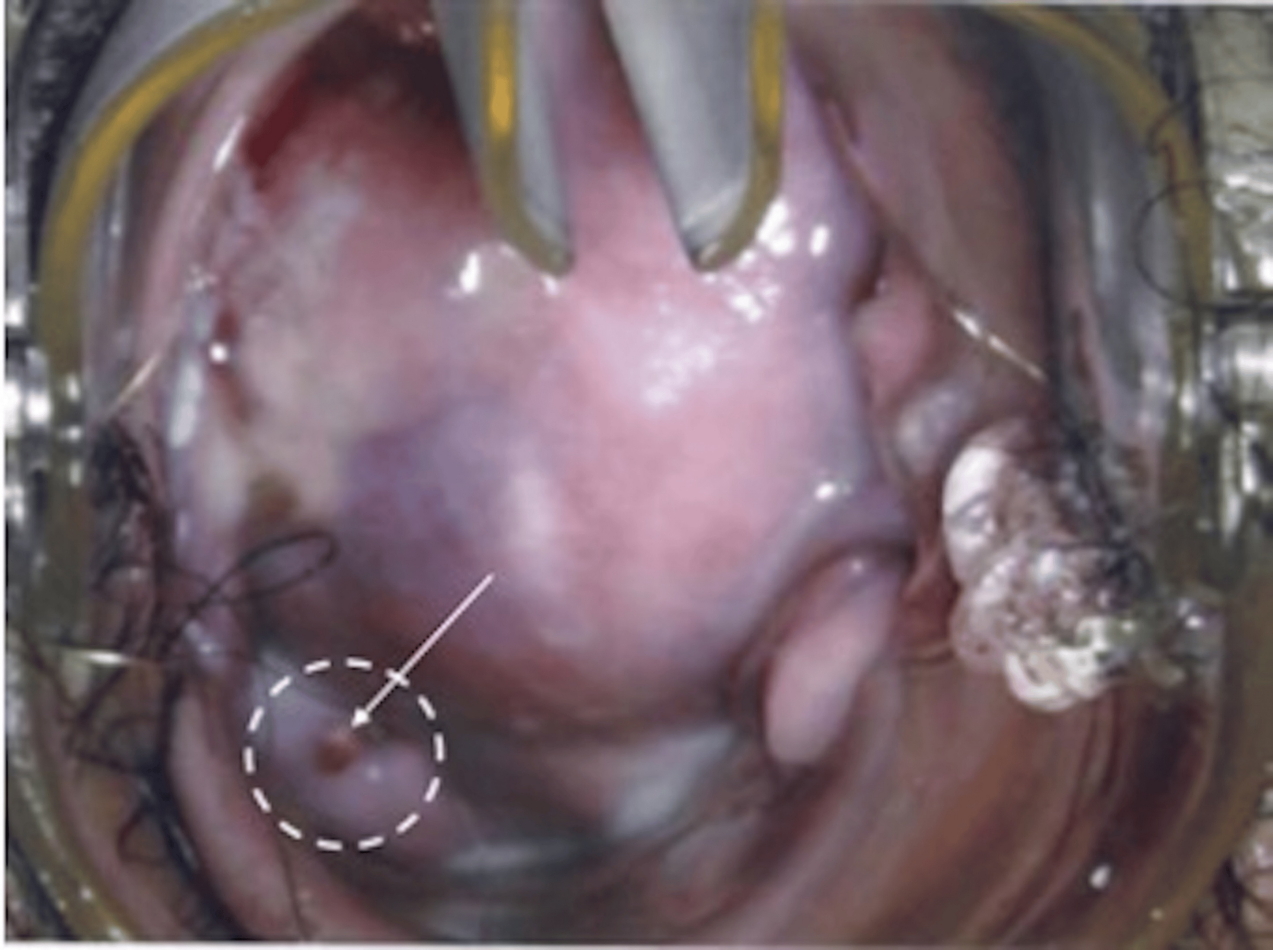
Mass arising from the posterior fornix measuring 1 cm on rectovaginal examination after three months of oral Dienogest.

On follow-up after three months of oral Dienogest treatment, she reported minimal side effects of irregular bleeding (minimal vaginal spotting one week after starting the medication), and she was subsequently amenorrhoeic. She also was symptom-free with no further postcoital bleeding and dysmenorrhoea. A rectovaginal examination demonstrated a smaller mass of approximately 1 cm in the posterior fornix (Figure 4).

**Figure 5:**
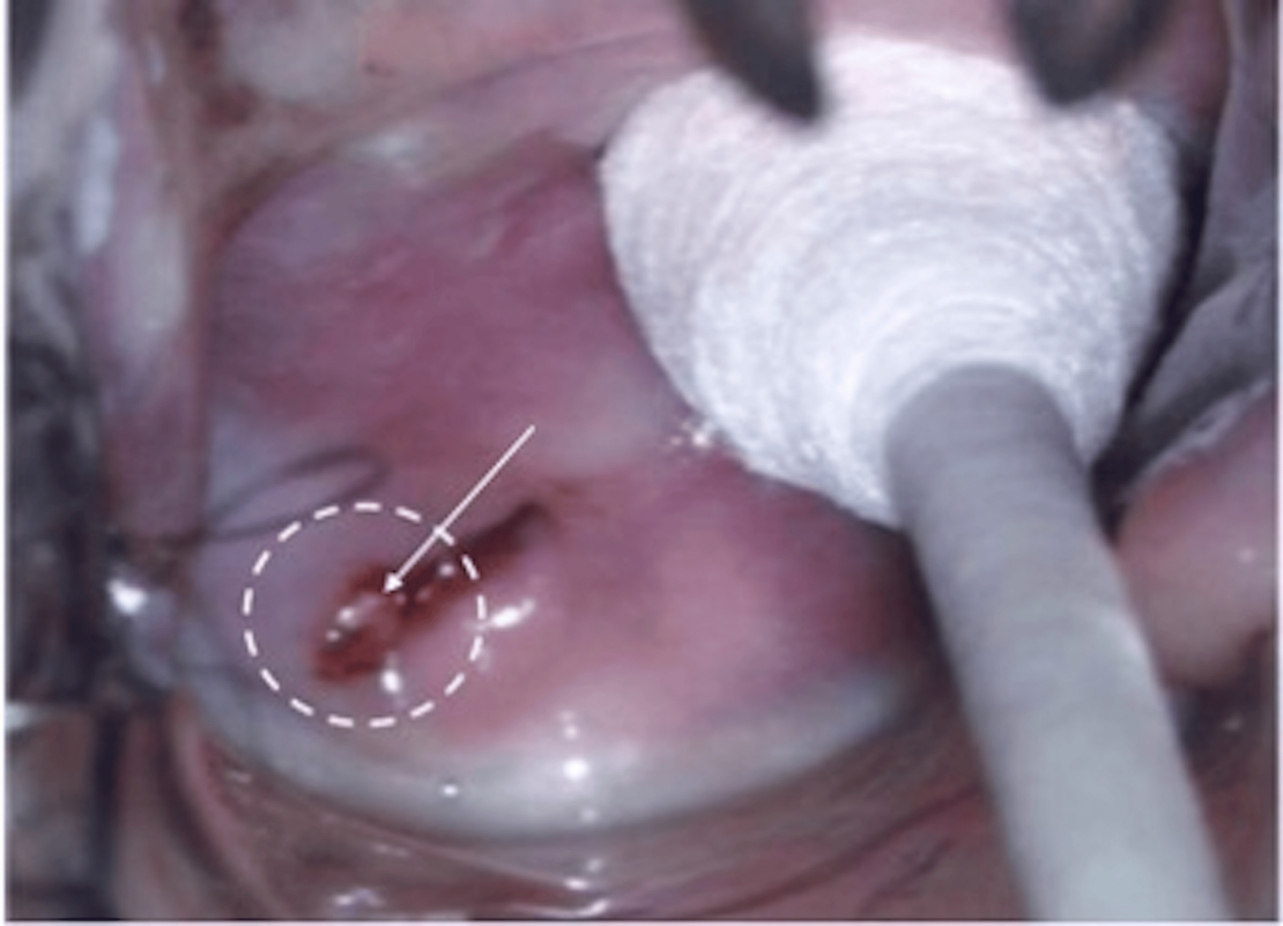
Mass arising from the posterior fornix measuring 0.5 cm during rectovaginal examination after six months of oral Dienogest.

**Figure 6:**
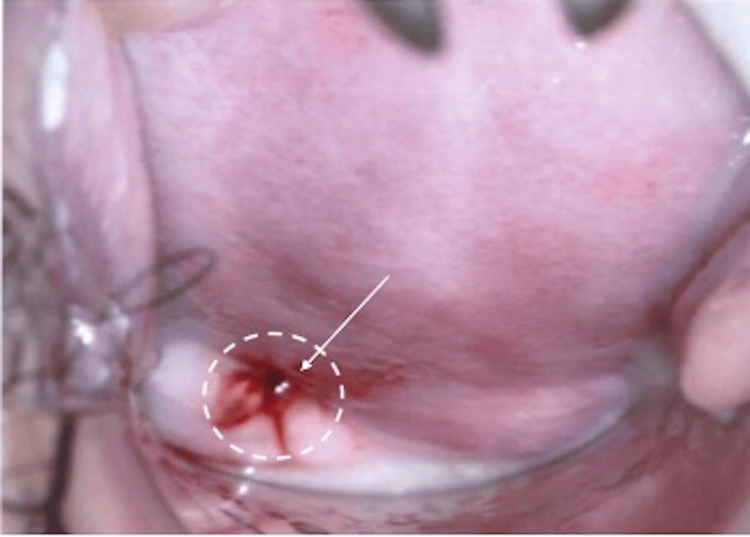
After nine months of oral Dienogest: remission of vaginal endometriosis.

**Figure 7:**
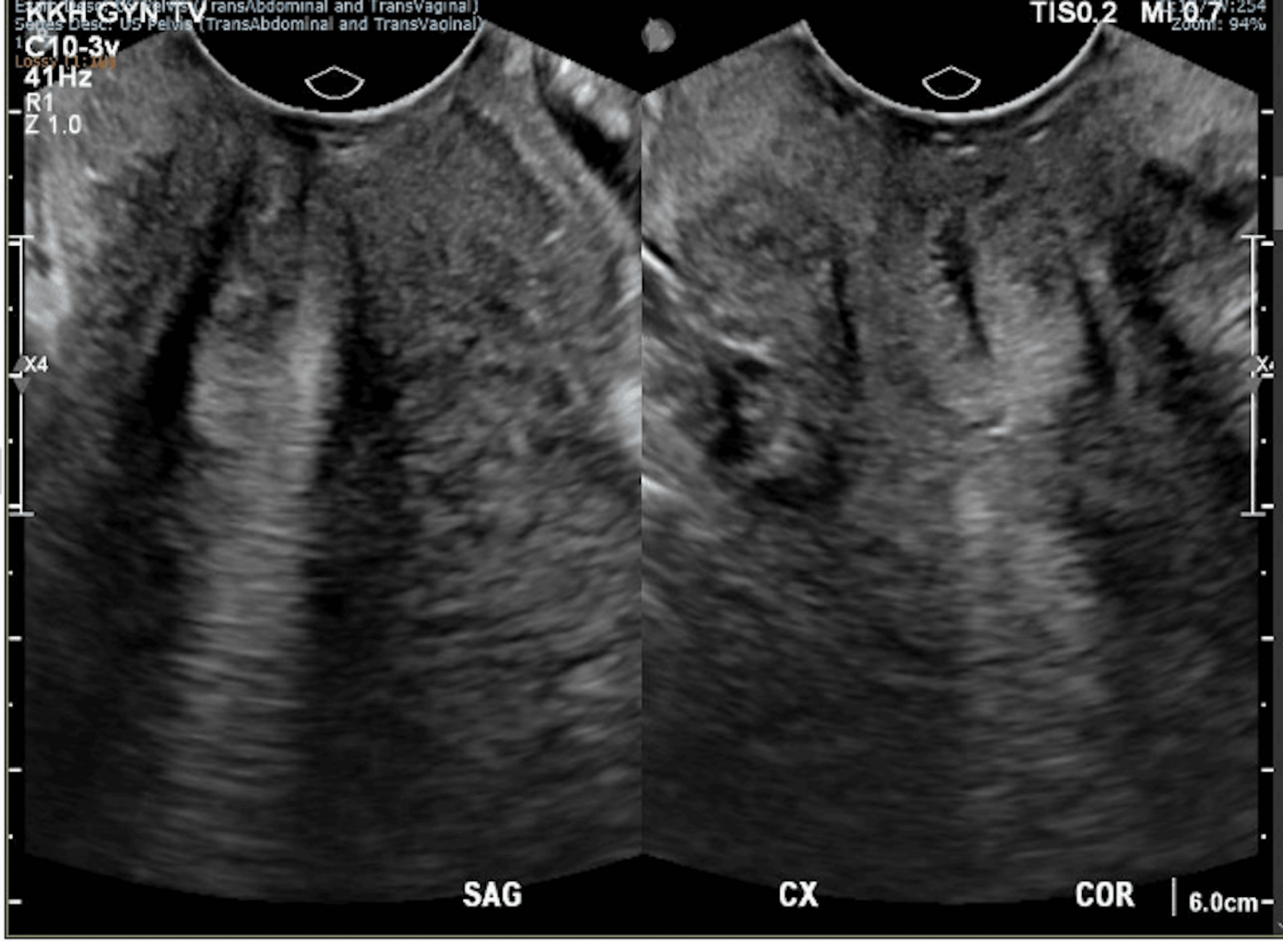
Sagittal (left) and coronal (right) views of posterior cervix on transvaginal ultrasonography post treatment showing resolution of vaginal endometriosis and endometriotic deposits in the Pouch of Douglas.

After six months of completing Dienogest, a rectovaginal examination showed that the mass measured 0.5 cm (Figure 5) and was barely visible in the posterior fornix. The patient also reported light abnormal uterine bleeding of two weeks in the middle of treatment. In March 2021, after nine months of therapy, pelvic ultrasonography and examination both demonstrated complete resolution of the vaginal endometriosis and endometriotic deposits in the Pouch of Douglas (Figures 6, 7). She also reported resolution of her prolonged menstruation. Continuing of the oral Dienogest treatment was agreed upon, and since June 2020, the patient has remained well and asymptomatic at two years with the physical appearance similar to that in Figure 6.

## Discussion

Rectovaginal endometriosis remains a therapeutic challenge, with limited studies on this topic and widely differing opinions on the treatment algorithm of DIE [6]. With no permanent cure, the treatment approach for DIE should be individualized. Treatment conundrums include relieving endometriotic-associated pain and improving fertility rates [1]. Demographics such as the patient’s age, symptoms, location of endometriotic deposits, and the patient’s fertility wishes and preferences are pivotal factors to consider.

This case illustrates the recurrence of vaginal endometriosis, possibly due to residual disease following initial surgery and a limited duration of hormonal therapy. Surgical resection can eliminate visible endometriosis lesions but is not a curative treatment. DIE, particularly in the rectovaginal space, is often challenging to excise completely, and microscopic lesions may persist despite surgical intervention [7], with a recurrence rate as high as 7% within two to six years after surgery [8].

Recurrence is common, at 40-50% at five years, especially when there is an influence of circulating oestrogens that fuel the growth of endometriotic foci [9]. This patient received postoperative gonadotropin-releasing hormone (GnRH) agonist therapy for seven months and Mirena for only five months before removal due to malposition, which may have limited the long-term suppressive effect. Notably, there was a 21-month gap without hormonal treatment, during which recurrence likely occurred, consistent with data showing high relapse rates in the absence of maintenance therapy [10].

DIE in the posterior fornix also carries a higher risk of recurrence due to its anatomical complexity and hormonal responsiveness [11]. The patient’s subsequent positive response to Dienogest, with sustained remission and lesion resolution, aligns with evidence supporting its efficacy and tolerability for long-term management [12].

Currently, medical therapies that are approved for endometriosis include non-hormonal, non-steroidal anti-inflammatory drugs and hormonal therapies such as GnRH agonists, combined oral contraceptives (COC), and progestogens [1]. Existing literature provide evidence for the success of various medical therapies of DIE involving the bladder and bowel using danazol, GnRH agonists, and low-dose COC [6]. However, available reports and series studying the use of Dienogest monotherapy for deep endometriosis [13] are limited, with only one demonstrating the efficacy of vaginal Dienogest in rectovaginal endometriosis [13].

Progestins approved for use vary between countries, including Dienogest, a relatively novel therapy for endometriosis. Dienogest is a progestin with highly selective progesterone activity that inhibits gonadotropin secretion, thereby reducing oestrogen levels and consequently causing the decidualization of endometrial tissue and atrophy of endometriotic lesions. Its effectiveness, safety, and tolerability profile as a long-term management strategy for endometriosis in routine clinical practice are increasingly being researched on a large scale [1].

In this case report, the use of Dienogest has proven to be effective in resolving the symptoms of pain and postcoital bleeding in three months. It has also shown to effect reduction and even complete remission of the endometriotic lesions after nine months of therapy, avoiding the need for repeat surgical intervention. This finding is congruent with existing evidence that shows the improvement of symptoms before the reduction in size of the lesion [2]. Further, Wu et al.'s recent meta-analysis involving 256 patients illustrated significant improvement in the relief of dysmenorrhoea, dyspareunia, and pelvic pain, besides rectosigmoid lesion size after Dienogest monotherapy in targeting DIE [3]. In a nine-year observational study involving 157 women, Maiorana et al. demonstrated progressive reduction in endometriotic-associated symptoms, mostly with dysmenorrhoea [14]. Favourable outcomes like these accentuate the need for more extensive research that can address the paucity of data that evaluate appropriate treatment strategies of DIE, specifically rectovaginal endometriosis [2], potentially redefining our current practice.

While several studies have supported the effectiveness of Dienogest in symptom control, there is a scarcity of literature demonstrating the efficacy of the drug on regression of endometriotic lesions in DIE, particularly rectovaginal endometriosis [2]. To our knowledge, available evidence is restricted to only case reports, most of which are on bladder endometriosis [15,16] and only one on vaginal endometriosis [6]. The variable response of lesions towards the treatment may also be dependent on the differing structural features of endometriotic lesions [6]. Heterogeneity in the outcomes of symptom improvement is also noted in the current evidence [1]. The proven complete resolution of the DIE lesion in this case, however, suggests the curative potential of Dienogest in DIE.

In the battle against endometriosis, a chronic progressive disease, the safety profile of long-term medical therapy is indubitably a crucial consideration. The most common adverse effect of Dienogest is observed to be irregular bleeding, known to be highly associated with discontinuation of the treatment [6]. According to a recent prospective cohort study conducted in Korea evaluating the safety profile of Dienogest for the treatment of endometriosis, abnormal uterine bleeding was commonly reported in short-term use of the drug. However, it tends to improve in terms of frequency and intensity with continued use of the treatment [17]. Other common adverse effects include headache, weight gain, breast tenderness, and acne. A promising outlook towards Dienogest is apparent from the international interest taken in further evaluation of the safety profile of Dienogest monotherapy and other hormonal alternatives for endometriosis. VIPOS (Visanne Post-approval Observational Study) is one such ongoing large-scale long-term cohort study that is in the works [5]. Nonetheless, Murji’s literature review, which summarized expert recommendations from various studies, reported that Dienogest does not pose significant additional risk of breast or other types of cancer compared with other progestins and COC [18].

Existing hormonal modalities, such as GnRH agonists, have been widely used and accepted as a treatment option for endometriosis. However, their long-term use is associated with high risk of hypoestrogenic side effects and significant decrease in bone mineral density if administered without hormonal replacement therapy. Conversely, Dienogest has demonstrated a favourable safety profile, as proven by high compliance and a low discontinuation rate secondary to adverse effects after prolonged use [14].

Further, the majority of patients with endometriosis are of reproductive age; fertility is therefore an important consideration when counselling patients on the long-term management of endometriosis. While Dienogest does not function as a contraceptive method, it provides complete ovulation inhibition if administered at 2 mg daily [19]. Pharmacological data revealed a prompt return to fertility with the rapid resumption of ovulation (ranging 1-43 days) upon cessation of Dienogest [20]. Notably, this case reinforces the importance of continuous hormonal suppression following surgery in patients with severe or deep endometriosis to reduce recurrence risk [10].

## Conclusions

DIE remains a treatment conundrum for both gynaecologists and patients, with significant risks complicating surgical intervention and excision of DIE. The continual development of imaging modalities and increasing awareness of endometriosis and its effect on the quality of life of affected women have inadvertently led to the increasing incidence of deep infiltrating endometriosis. While Dienogest is becoming more widely accepted as one of the mainstay medical management options for DIE, there still remains a scarcity in studies assessing its efficacy and its adverse reactions in the long-term in the treatment of specifically rectovaginal endometriosis. The above lessons gleaned from this experience reinforce the need for greater sharing of outcomes and experiences of rare presentations of DIE, allowing future systematic review and reliable conclusions regarding different treatment modalities for these rare scenarios.

## References

[1] Becker CM, Bokor A, Heikinheimo O (2022). ESHRE guideline: endometriosis. Hum Reprod Open.

[2] Leonardo-Pinto JP, Benetti-Pinto CL, Cursino K, Yela DA (2017). Dienogest and deep infiltrating endometriosis: the remission of symptoms is not related to endometriosis nodule remission. Eur J Obstet Gynecol Reprod Biol.

[3] Wu H, Liu JJ, Ye ST, Liu J, Li N (2024). Efficacy and safety of dienogest in the treatment of deep infiltrating endometriosis: a meta-analysis. Eur J Obstet Gynecol Reprod Biol.

[4] Techatraisak K, Hestiantoro A, Ruey S (2019). Effectiveness of dienogest in improving quality of life in Asian women with endometriosis (ENVISIOeN): interim results from a prospective cohort study under real-life clinical practice. BMC Womens Health.

[5] Heinemann K, Imthurn B, Marions L, Gerlinger C, Becker K, Moehner S, Faustmann T (2020). Safety of dienogest and other hormonal treatments for endometriosis in real-world clinical practice (VIPOS): a large noninterventional study. Adv Ther.

[6] Agarwal S, Fraser MA, Chen I, Singh SS (2015). Dienogest for the treatment of deep endometriosis: case report and literature review. J Obstet Gynaecol Res.

[7] Vercellini P, Carmignani L, Rubino T, Barbara G, Abbiati A, Fedele L (2009). Surgery for deep endometriosis: a pathogenesis-oriented approach. Gynecol Obstet Invest.

[8] Koga K, Takamura M, Fujii T, Osuga Y (2015). Prevention of the recurrence of symptom and lesions after conservative surgery for endometriosis. Fertil Steril.

[9] Cheong Y, Tay P, Luk F, Gan HC, Li TC, Cooke I (2008). Laparoscopic surgery for endometriosis: How often do we need to re-operate?. J Obstet Gynaecol.

[10] Guo SW (2009). Recurrence of endometriosis and its control. Hum Reprod Update.

[11] Bazot M, Darai E, Hourani R, Thomassin I, Cortez A, Uzan S, Buy JN (2004). Deep pelvic endometriosis: MR imaging for diagnosis and prediction of extension of disease. Radiology.

[12] Strowitzki T, Faustmann T, Gerlinger C, Seitz C (2010). Dienogest in the treatment of endometriosis-associated pelvic pain: a 12-week, randomized, double-blind, placebo-controlled study. Eur J Obstet Gynecol Reprod Biol.

[13] Ebert AD (2018). Daily vaginal application of dienogest (Visanne©) for 3 months in symptomatic deeply infiltrating rectovaginal endometriosis: a possible new treatment approach?. Case Rep Obstet Gynecol.

[14] Maiorana A, Maranto M, Restivo V, Gerfo DL, Minneci G, Mercurio A, Incandela D (2024). Evaluation of long-term efficacy and safety of dienogest in patients with chronic cyclic pelvic pain associated with endometriosis. Arch Gynecol Obstet.

[15] Harada M, Osuga Y, Izumi G (2011). Dienogest, a new conservative strategy for extragenital endometriosis: a pilot study. Gynecol Endocrinol.

[16] Takagi H, Matsunami K, Ichigo S (2011). Novel medical management of primary bladder endometriosis with Dienogest: a case report. Clin Exp Obstet Gynecol.

[17] Cho B, Roh JW, Park J (2020). Safety and effectiveness of dienogest (Visanne®) for treatment of endometriosis: a large prospective cohort study. Reprod Sci.

[18] Murji A, Biberoğlu K, Leng J, Mueller MD, Römer T, Vignali M, Yarmolinskaya M (2020). Use of dienogest in endometriosis: a narrative literature review and expert commentary. Curr Med Res Opin.

[19] Andres Mde P, Lopes LA, Baracat EC, Podgaec S (2015). Dienogest in the treatment of endometriosis: systematic review. Arch Gynecol Obstet.

[20] Klipping C, Duijkers I, Faustmann T (2010). Pharmacodynamic study of four oral dosages of dienogest. Fertil Steril.

